# In vitro characterization and in vivo toxicity, antioxidant and immunomodulatory effect of fermented foods; Xeniji™

**DOI:** 10.1186/s12906-017-1845-6

**Published:** 2017-06-30

**Authors:** Noraisyah Zulkawi, Kam Heng Ng, Rizi Zamberi, Swee Keong Yeap, Dilan Satharasinghe, Indu Bala Jaganath, Anisah Binti Jamaluddin, Sheau Wei Tan, Wan Yong Ho, Noorjahan Banu Alitheen, Kamariah Long

**Affiliations:** 1 Elken Sdn Bhd, 20, Bangunan Elken, Jalan 1/137C, Batu 5, Jalan Kelang Lama, 58000 Kuala Lumpur, Malaysia; 2 Institute of Bioscience, Universiti Putra Malaysia, Serdang, Selangor Malaysia; 3 Biotechnology Research Centre, Malaysian Agricultural Research and Development Institute (MARDI), 43400 Serdang, Selangor Malaysia; 4 China-ASEAN College of Marine Sciences, Xiamen University Malaysia, Jalan Sunsuria, Bandar Sunsuria, 43900 Sepang, Selangor Malaysia; 5 Department of Basic Veterinary Sciences, Faculty of Veterinary Medicine & Animal Science, University of Peradeniya, Peradeniya, 20400 Sri Lanka; 6 School of Biomedical Sciences, The University of Nottingham Malaysia Campus, 43500 Semenyih, Selangor Malaysia; 7 Department of Cell and Molecular Biology, Faculty of Biotechnology and Biomolecular Science, Universiti Putra Malaysia, 43400 Serdang, Selangor Malaysia

**Keywords:** Lactic acid bacteria, Yeast, Organic acid, Antioxidant, T cells

## Abstract

**Background:**

Xeniji, produced by fermenting various types of foods with lactic acid bacteria and yeast, has been commonly consumed as functional food. However, nutrition value, bioactivities and safety of different fermented products maybe varies.

**Methods:**

Organic acid and antioxidant profiles of Xeniji fermented foods were evaluated. Moreover, oral acute (5 g/kg body weight) and subchronic toxicity (0.1, 1 and 2 g/kg body weight) of Xeniji were tested on mice for 14 days and 30 days, respectively. Mortality, changes of body weight, organ weight and serum liver enzyme level were measured. Liver and spleen of mice from subchronic toxicity study were subjected to antioxidant and immunomodulation quantification.

**Results:**

Xeniji was rich in β-carotene, phytonadione, polyphenol, citric acid and essential amino acids. No mortality and significant changes of body weight and serum liver enzyme level were recorded for both oral acute and subchronic toxicity studies. Antioxidant level in the liver and immunity of Xeniji treated mice were significantly upregulated in dosage dependent manner.

**Conclusion:**

Xeniji is a fermented functional food that rich in nutrients that enhanced antioxidant and immunity of mice.

**Graphical abstract:**

Xeniji that rich in β-carotene, phytonadione, polyphenol, citric acid and essential amino acids promote antioxidant and immunity in mice without causing toxic effect.
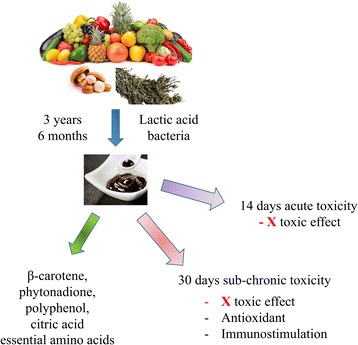

**Electronic supplementary material:**

The online version of this article (doi:10.1186/s12906-017-1845-6) contains supplementary material, which is available to authorized users.

## Background

Rapid expansion of scientific study has shown the role of diet in preventing and controlling chronic diseases particularly non-communicable diseases. Population who practice diet that rich in fruits and vegetables are recorded with lower rate of chronic diseases [1]. This effect maybe contributed by the polyphenols that present in the fruits and vegetables. Polyphenols are antioxidants that scavenged oxidative stress in the cells. The polyphenols in fruits and vegetables have been reported with various properties including antioxidant, anti-tumor and protection against cardiovascular disease [2]. Although fresh fruits and vegetables are good source of polyphenols [2], short shelf life and convenience to the consumers have limit the consumption of fruits and vegetables. Fermentation is one of the commonly used food processing methods to preserve and prolong shelf life for foods and vegetables. Moreover, fruits and vegetables fermented by yeast or lactic acid bacteria were also reported with enhance polyphenols and antioxidants [3, 4]. Thus, fermented fruits and vegetables are functional foods that may exert positive effects on maintenance of overall good health [5].

Fermented natural food is a common functional food in Japan produced by fermenting fruits and vegetable using lactic acid bacteria and yeast [6]. It was reported as a convenient functional food to supply sufficient and more digestible dietary nutrients [7]. In Japan, fermented foods, such as plant-based paste, Manda and Xeniji, that produced by fermenting various food ingredients including fruits, vegetables, herbs, mushrooms, seaweed, pulse, cereals with lactic acid bacteria have been widely consumed as health supplement [6, 8]. Xeniji is one of the commercial available fermented food that commonly consumed in Japan and Malaysia. However, the nutritional value, in vivo toxicity and bioactivities of Xeniji were yet to be reported. Although it is fermented using similar food ingredients and lactic acid bacteria as other commercial available fermented food, it is important to profile its nutrients as different types of fermented products may possess different level of antioxidant when fermenting by different source of carbohydrate [9]. In addition, over consumed of functional foods that rich in polyphenols may associate with some mild adverse effects [2]. Thus, it is important to profile the nutrients of the Xeniji. Previous study on other fermented food were carried out using rodent model [8, 10–15]. This study has also been validated in the in vivo mice model regarding safety and efficacy of Xeniji.

## Methods

### Preparation of fermented food

Commercial available Xeniji™ was provided by Elken Sdn Bhd, Malaysia. It was prepared by fermenting multiple food ingredients using commercial lactic acid bacteria (Table 1) for a period of 3 years and 6 months. Then, 1 g of Xeniji was weighed and mixed with the 1 mL of water followed by shaking at 400 rpm for 1 h. The sample was then centrifuged for 10 min 10,000 rpm at room temperature and the supernatant was recovered. The supernatant was concentrated using the rotary evaporator for further analysis.

**Table 1:** Materials of fermented fruits and vegetables, Xeniji™

	Material name	Content
Sugar	Brown sugar, Galacto-oligosaccharide (GOS) and Oligosaccharide	67.2%
Fruits	*Prunus domestica L.* (Prune), *Fragaria x ananassa* (Strawberry), *Malus domestica* (Apple), *Vitis pione* (Grape), *Prunus persica* (Peach), *Citrus unshiu* (Mandarin orange), Mulberry, Cherry blossom paste, *Citrus junos* (Yuzu), *Diospyros kaki* (Persimmon), *Actindia chinensis* (Kiwi), *Fortunella japonica* (Kumquat)*, Citrus limon* (Lemon), *Vaccinium corymbosum* (Blueberry), *Myrica rubra* (Artubus), *Pyrus pyrifola* (Pear), *Prunus mume* (Ume), *Citrus iyo* (Iyo-orange), *Ficus carica* (Fig), *Rubus buergeri* (Raspberry) and *Rubus fruticosus* (Blackberry)	18.0%
Vegetables and wild herbs	*Angelica keiskei* (Folium) (Angelica keiskei leaf powder), *Perilla frutescens* (Perilla), *Cucurbita maxima* (Pumpkin), *Raphanus sativus* (Japanese radish), *Spinacia oleracea* (Spinach), *Daucus carota* var. Sativus (Carrot), *Brassica oleracea*, acephala (Kale), *Hordeum vulgare* L. (Barley grass), *Corchorus olitorius* (Jew’s mallow), *Lycopersicon esculentum* (Tomato), *Cucumis sativus* (Cucumber), *Plantago asiatica* (Plantain), *Sasa veitchii* (Stripped bamboo), *Equisetum arvense* (Field horsetail), *Eriobotrya japonica* (Loquat leaf), *Brassia oleracea* var. Capitata (Cabbage), *Salanum melongena* (Eggplant), *Apium graveolens var. Dulce* (Celery), *Capsicum annuum* (Sweet pepper), *Mormodica charantia* (Bitter melon), *Brassica rapa* chinensis (Bok Choi), *Nelumbo nucifera* (Radix) (Lotus root), *Curcuma longa* (Turmeric), *Brassica oleracea var. italica* (Broccoli), *Zingiber officinale* (Ginger), *Petroselinum crispum* cv (Parsley), *Asparagus officinalis* var. Altilis (Asparagus) and *Oentanthe stolonifera* (Japanese Parsley)	7.4%
Mushrooms	*Ganoderma lucidum* (Reishi), *Lentinula edodes* (Shiitake mushroom), *Auricularia polytricha* (Jew’s ear), *Grifola frondosa* (Maitake mushroom)	1.3%
Seaweed	*Ascophyllum nodosum* (Kelp), *Laminaria japonica Areschoug* (Kombu), *Undaria pinnatifida suringer* (Wakame), *Fucus evanescens* (Fucus), *Sargassum fusiforme setchell* (Hijiki)	1.6%
Pulse and Cereals	*Glycine max* (Soybean), *Theobroma cacao* (Cocoa), *Zea mays L.* (Sweet Corn), *Oryza sativa* (Rice)	4.4%
Lactic acid bacteria species	*Lactobacillus brevis, Lactobacillus casei, Lactobacillus curvatus, Lactobacillus paracasei, Lactobacillus pentosus, Lactobacillus plantarum, Lactococcus lactis, Leuconostoc mesenteroides, Pediococcus acidilactici, Pediococcus pentosaceus*	0.1%

### Total phenolic content (TPC) of Xeniji

Total phenolic content of Xeniji was performed using Folin-Ciocalteu methodology according to the method by Thaipong et al. [16]. Briefly, 1 mL of the extracts were added with 5 mL of diluted Folin-Ciocalteu reagent and 4 mL of the 7.5% sodium carbonate solution further kept for 2 h at room temperature in dark. After that, absorbance was measured at 725 nm using spectrophotometer (Eppendorf, USA). Gallic acid (Sigma-Aldrich, USA) was used to generate standard curve and the results were expressed as μg gallic acid equivalent (GAE)/g extract.

### Total flavonoid content (TFC)

Total flavonoid content of Xeniji was determined by the aluminum chloride colorimetric method [17]. In brief, 1 mL of extract was mixed with 4 mL distilled water and then, 0.3 ml 10% AlCl_3_ solution was added after 5 mins of incubation and the mixture was allowed to stand for 6 mins. Then, 2 mL of 1 N NaOH solution was added and the final volume of the mixture was brought to 10 mL with distilled water. The mixture was allowed to stand for 15 mins and the absorbance was measured at 510 nm. The TFC was calculated from a calibration curve and the result was expressed as mg quercetin equivalent/gram extract.

### Ferric reducing antioxidant potential (FRAP)

The FRAP assay was done according to Mohamad et al. [18] with some modifications. The fresh working solution was prepared by mixing 25 mL of 300 mM acetate buffer (pH 3.6), 2.5 mL of 10 mM TPTZ solution, and 2.5 mL of 20 mM FeCl_3_.6H_2_O solution. The sample extract (150 uL) was allowed to react with 2.85 mL of the FRAP solution for 30 min in the dark condition. Readings of the coloured product (ferrous tripyridyltriazine complex) were then taken at 593 nm. Results were expressed in mg ascorbic acid equivalent (AAE)/g extract.

### Reverse phase chromatography profiling of organic acids

The organic acids were separated and quantified by reversed phase chromatography with external calibration graphs (Table 2). Organic acids in the sample were separated on an Extrasil ODS column (250 mm × 4.6 mm, 5 μm) and the detector was set at λ = 210 nm and λ = 245 nm. Determination of organic acids was carried out at isocratic conditions at 45 °C, using a mobile phase of 50 mM phosphate solution (6.8 g potassium dihydrogen phosphate in 900 mL water, pH 2.8). The flow rate of the mobile phase was set at 0.7 mL/min.

**Table 2:** Retention time, concentration ranges of lineal response and correlation coefficient for standard organic acids

Organic acid standard	Rt (min)	Concentration ranges (ppm)	Equation	R^2^
Oxalic	3.884	1750–350	Y = 7585.1×-72,810	0.9937
Tartaric	4.148	5000–1000	Y = 2914.9×-2758	0.9942
Ascorbic	5.295	1750–350	Y = 8327.7×-7497.5	0.9921
Lactic	5.857	15,000–3000	Y = 769.58× + 4607	0.9939
Acetic	6.334	10,000–2000	Y = 253.82× + 2571	0.9991
Citric	6.953	10,000–2000	Y = 405.19× + 1826.9	0.9953
Succinic	9.052	10,000–2000	Y = 428.23× + 321.26	0.9936
Kojic	11.31	500–100	Y = 56,358×-22,343	0.9913

### Ultra-performance liquid chromatography profiling of amino acid

Amino acids (9 essential and 9 none-essential amino acids) and gamma amino butyric acids (GABAs) contents were determined using ultra-performance liquid chromatography (UPLC) as described by Koh et al., [19]. Extracts of fermented paste were filtered with 0.22 μM nylon syringe filter. Then, a total of 10 μL filtrated fermented pastes were further derivatized with 70 μL of AccQ-TagTM Ultra borate buffer and mixed vigorously. Then, 20 μL of AccQTM Fluor reagent was added, and the mixture was vortexed for a while before being heated at 55 °C for 10 min, followed by 1 μL injection of the mixture into the UPLC system. The amino acid profile of fermented paste was obtained using AccQ-TagTM Ultra column (2.1 mm × 100 mm, 1.7 μM) at a flow rate of 0.7 mL/min with column temperature controlled at 55 °C under the UV spectra of 260 nm. The gradient elution consists of AccQ-TagTM Ultra Eluent A (ACN:FA:Ammonium;10:6:84) and AccQ-TagTM Ultra Eluent B (ACN:FA;98:2). Gradient elution was conducted as follows: 0 to 0.54 min, maintained at 99.9% A; 0.54 to 5.74 min, linear gradient from 99.9 to 90.9% A; 5.74 to 7.74 min, linear gradient from 90.9 to 78.8% A; 7.74 to 8.50 min, linear gradient from 78.8 to 40.4% A and hold for 0.3 min at 40.4% A; 8.80 to 8.90 min, linear gradient from 40.4 to 99.9% A and then maintained at 99.9% for another 2.1 min. All analyses were performed in triplicates. The UPLC profile, which was used for the standardization of the samples throughout the study is shown in Additional file 1: Fig. S1.

### High-performance liquid chromatography profiling of vitamin

Vitamin-A (beta carotene and retinol), −B1, −B2, −B3, −B5, −B6, −B7, −B9, −C, −D2, −D3, −E (alpha-tocopherol) and K1 were detected using HPLC by qualified laboratory testing company (ALS Technichem (M) Sdn Bhd, Malaysia).

### Animal

The study was conducted according to the guidelines and was approved by the Institution of Animal Care and Use Committee (IACUC), Universiti Putra Malaysia (UPM/IACUC/AUP-R097/2014). A total of 26 male Balb/c mice (aged 6 weeks old) were purchased from animal house, Institute of Bioscience, Universiti Putra Malaysia (UPM). Both acute and subchronic toxicity studies were started when the animal were acclimatised until 8 weeks old. The mice were house in plastic cage (*n* = 3), using commercial sawdust bedding, fed with distilled water and standard pellets (Crude protein 21%, crude fibre 5%, crude fat 3%, moisture 13%, ash 8%, calcium 0.8%, phosphorus 0.4%) (Gold Coin, Malaysia) ad libitum under housing condition 22 °C, 12 h of day/dark light cycles.

### Acute toxicity test

Male Balb/c mice (*n* = 6) at weight approximate 21 g were separated into 2 groups; which are normal control that fed once with water (*n* = 3) and treated group fed with 5 g/kg body weight of plant-based fermented paste (Xeniji 5.0) (*n* = 3) by oral gavage using stainless steel gavage needle with volume 0.20 mL. The mice were monitor until day 14, recorded for body weight, anesthetised with isoflurane, and euthanised by cervical dislocation. Then, kidney, liver and spleen weight were recorded, serum was collected and liver enzyme markers: aspartate aminotransferase (AST), alanine aminotransferase (ALT) and alkaline phosphatase (ALP) were evaluated using Hitachi 902 Automatic Analyzer (Hitachi, Japan) using reagents from Roche (Germany).

### Subchronic toxicity test

Male Balb/c mice (*n* = 20) at weight approximate 21 g were separated into 4 groups (*n* = 5 per group); which are normal control that oral fed daily with water and treated groups oral fed daily with either 0.1, 1.0 or 2.0 g/kg body weight of plant-based fermented paste by oral gavage using stainless steel gavage needle with volume 0.20 mLAfter 30 days of treatment, body weight was recorded and all mice were anesthetised with isoflurane, and euthanised by cervical dislocation. Then, serum, kidney, liver and spleen were collected from normal and Xeniji treated mice, washed and separated into 2 parts. One part of liver and spleen were stored in RNA later for quantitative real time PCR (qRT-PCR) assay, while another part was meshed using 70 μm cell strainer (SPL, Korea) to prepare liver and spleen homogenate [18] for liver antioxidant quantification and T/NK cell immunophenotyping, respectively. Spleen was washed initially with lysis buffer (8 g NH_4_Cl, 1 g Na_2_EDTA, 0.1 g KH_2_PO_4_, pH 7.4) to remove red blood cells and finally with PBS to clean splenocytes [20].

### Serum biochemical analysis

Serum was collected and liver enzyme markers: aspartate aminotransferase (AST), alanine aminotransferase (ALT) and alkaline phosphatase (ALP) were evaluated using Hitachi 902 Automatic Analyzer (Hitachi, Japan) using reagents from Roche (Germany).

### Quantitative real time PCR (qRT-PCR) of antioxidant genes in liver and cytokines in spleen

Total RNA from the liver and spleen were extracted using RNeasy mini kit (Qiagen, USA) according to the manufacturer’s protocol. The concentration and purity of extracted RNA were measured Biospectrometer (Eppendorf, Hamburg, Germany), and cDNA was subsequently synthesized from 1 μg of total RNA using NEXscript cDNA synthesis kit (NEX Diagnostics, Korea) according to the manufacturer’s protocols. Primers for target genes GSTA2, GCLM [21], IL-2, IL-18, and house-keeping gene β-actin [21] were listed in Table 3. Quantitative real time PCR (qRT-PCR) was performed using NEXpro qPCR Evagreen Master Mix (NEX Diagnostics, Korea) by CFX96 Real Time PCR system (Biorad, USA) with the following steps: 95 °C for 2 min, 40 cycles of 95 °C for 10 s, 60 °C for 45 s and acquisition of fluorescent signal. The specificity and efficiency of primers were confirmed by qRT-PCR melt curve and standard curve analyses (Additional file 2: Fig. S2). Expression of target genes in the treatment groups and control group was normalised using the β-actin and the fold change in the expression of each target gene was calculated by the efficiency-corrected method using ratio = 2^-ΔΔCq^ [21].

**Table 3:** Primer sequences for qRT-PCR gene expression study

Gene	Primer sequence (5′–3′)	
	Forward	Reverse
GCLM	AATCAGCCCCGATTTAGTCAGG	CCAGCTGTGCAACTCCAAGGAC
GSTA2	CGCCACCAAATATGACCTCT	CCTGTTGCCCACAAGGTAGT
IL-2	TGAGTCAGCAACTGTGGTGG	GCCCTTGGGGCTTACAAAAAG
IL-18	GGGCACCCTAGCTCATGTTT	GCACAAGACGTGTGAGGAGA
β-actin	TCCTTCCTGGGCATGGAG	AGGAGGAGCAATGATCTTGATCTT

### Liver antioxidant and nitric oxide (NO) quantification

Antioxidant level in the liver of normal control and Xeniji treated mice were determined by evaluating FRAP, GSH, MDA and NO quantification. FRAP and MDA assays were performed according to the previously published method [18]. In addition, GSH and NO levels in the liver were quantified using Glutathione assay kit (Sigma-Aldrich, USA) and Griess reagent (Invitrogen, USA) according to the manufacturers’ protocols.

### Spleen T and NK cell immunophenotyping

Harvested splenocytes were stained with either 1 μg of CD3-FITC (AB24948, Abcam, USA), CD4-PE (AB86859, Abcam, USA) and CD8-Cy5 (AB39850, Abcam, USA) or 1 μg of CD3-FITC (AB24948, Abcam, USA) and NK1.1-APC (AB25352, Abcam, USA) for 30 min. Then, splenocytes were washed twice with PBS and subjected to flow cytometry analysis using BD FACSCalibur (BD, USA).

### Serum IL-2, IL-12, IL-18 and IFN-γ quantification

IL-2, IL-12, IL-18 and IFN-γ cytokines levels in the serum were tested using ELISA kits according to manufacturer’s protocol (R&D system, USA).

### Statistical analysis

Means and standard deviation from five mice per groups (each with three technical replicates) were calculated using excel for all experiments. Significant difference (*p* < 0.05) between the normal and Xeniji treated groups for all experiments was tested using one way analysis of variance (ANOVA) followed by post-hoc Duncan analysis using SPSS version 20.

## Results

### Antioxidant, organic acids and amino acids profiles of Xeniji

Xeniji fermented food was recorded with higher total phenolic content (5.12 ± 0.02 mg GAE/g extract) and total flavonoid content (1.18 ± 0.01 mg QE/g extract). Present of these antioxidant polyphenol and flavonoid have contributed to in vitro antioxidant activity, as detected by FRAP assay (Table 4). On the other hand, citric acid was the highest organic acid detected in Xeniji. This was followed by succinic acid, acetic acid and oxalic acid, which were approximately 10 times lower concentration than citric acid. In terms of amino acid profile, Xeniji was detected with all nine essential amino acid (total concentration of essential amino acid: 65.25 mg/100 g Xeniji), seven none-essential amino acid (total concentration of none-essential amino acid: 27.05 mg/100 g Xeniji) and moderate level of nonprotein amino acid GABA (0.50 mg/100 g Xeniji). Total concentration of essential amino acid in Xeniji was 2.4 times higher than none-essential amino acid, with aspartic acid (28 mg/100 g Xeniji) was recorded as the most highly detected amino acid. Xeniji contains high amount of vit-A (5050 μg/100 g samples). In addition, vit-B9, vit-c, vit-D3 and vit-K1 were also detected in Xeniji.

**Table 4:** Antioxidant, organic acids and amino acids profiles of Xeniji

		Xeniji water extract
Antioxidant profile	Total phenolic content (TPC)	5.12 ± 0.02
	(mg GAE/g extract)	
	Total flavonoid content (TFC)	1.18 ± 0.01
	(mg QE/g extract)	
	FRAP	3.30 ± 0.01
	(mg AAE/g extract)	
Organic acids profile (mg/g sample)	Oxalic acid	2.69 ± 0.07
	Lactic acid	1.02 ± 0.22
	Acetic acid	2.96 ± 0.32
	Citric acid	31.01 ± 1.40
	Succinic acid	3.55 ± 0.20
	Kojic acid	0.04 ± 0.001
Amino acids (mg/100 g samples)	Alanine^*a*^	8.50 ± 0.02
	Arginine^*a*^	0.65 ± 0.07
	Aspartic acid^*a*^	28.00 ± 0.14
	Cysteine^*a*^	0.55 ± 0.03
	Glutamic acid^*a*^	1.85 ± 0.07
	Glycine^*a*^	8.20 ± 0.28
	Proline^*a*^	13.25 ± 0.12
	Serine^*a*^	0.65 ± 0.02
	Tyrosine^*a*^	3.60 ± 0.14
	Asparagine^*b*^	n.d.
	Glutamine^*a*^	n.d.
	Histidine^*b*^	n.d.
	Isoleucine^*b*^	5.75 ± 0.71
	Leucine^*b*^	6.75 ± 0.02
	Lysine^*b*^	0.90 ± 0.14
	Methione^*b*^	0.85 ± 0.06
	Phenylalanine^*b*^	3.40 ± 0.28
	Threonine^*b*^	1.05 ± 0.07
	Tryptophan^*b*^	n.d.
	Valine^*b*^	8.35 ± 0.71
Nonprotein amino acid (mg/100 g samples)	Gamma amino butyric acid (GABA)	0.50 ± 0.01
Vitamin (μg/100 g samples)	A (β-carotene)	5050.00
	A (Retinol)	n.d.
	B1	n.d.
	B2	n.d.
	B3	n.d.
	B5	n.d.
	B6	n.d.
	B7	n.d.
	B9	2.20
	C	13.70
	D2	n.d.
	D3	10.60
	E (Alpha-tocopherol)	n.d.
	K1 (Phytonadione)	74.70

^a^Non-essential amino acids (NEAA); ^b^Essential amino acids (EAA); ± standard error; n.d. = not detected

### Acute toxicity test

Although Xeniji was rich in nutrients, before evaluated the in vivo bioactivities, the in vivo acute and subchronic toxicity were performed to confirm safety of this product. Acute toxicity test at 5 g/kg body weight (BW) did not observe with mortality, sign and symptom of toxicity, changes of body weight (BW) and organ weight (Table 5) throughout the 14 days of incubation period. In addition, biochemical analysis on serum liver enzyme profile (AST, ALT and ALP) reveal that there were no significant differences (*p* < 0.05) between the normal control and Xeniji 5 g/kg BW fed mice (Table 5).

**Table 5:** Body weight, organ weight and serum liver enzyme profile of normal and 5 g/kg body weight of Xeniji treated mice for acute toxicity study

	Normal	Xeniji (5 g/kg BW)
Day 0 BW (g)	20.21 ± 1.23	22.53 ± 1.29
Day 14 BW (g)	21.06 ± 0.73	23.02 ± 1.86
Liver weight (g)	1.41 ± 0.09	1.59 ± 1.86
Kidney weight (g)	0.51 ± 0.03	0.53 ± 0.04
Spleen weight (g)	0.14 ± 0.02	0.21 ± 0.01
Liver/BW ratio	0.0517	0.0537
Kidney/BW ratio	0.0187	0.0179
Spleen/BW ratio	0.0049	0.0069
ALT (U/L)	66.83 ± 2.33	63.40 ± 3.59
ALP (U/L)	69.75 ± 3.41	75.10 ± 6.77
AST (U/L)	108.32 ± 3.79	112.87 ± 3.41
Creatinine (μmol/L)	46.00 ± 3.56	46.00 ± 2.67

No significant different was observed at *p* < 0.05 and *p* < 0.001

### Subchronic toxicity test

For subchronic toxicity test, mice were orally fed with 0.1 g, 1.0 g or 2.0 g/kg BW of Xeniji daily for 30 days. After the treatment period, all Xeniji treated mice were survived without sign and symptom of toxicity, changes of body weight (BW), organ weight and serum liver enzyme profile were observed (Table 6).

**Table 6:** Body weight, organ weight and serum liver enzyme profile of normal and 5 g/kg body weight of Xeniji treated mice for 30 days of subchronic toxicity study

	Normal	Xeniji 0.1	Xeniji 1.0	Xeniji 2.0
Day 0 BW (g)	20.42 ± 0.125	18.96 ± 0.271	19.96 ± 1.297	19.67 ± 1.145
Day 28 BW (g)	24.52 ± 1.370	22.91 ± 1.023	23.93 ± 1.837	23.52 ± 1.116
BW gain (g)	4.10 ± 1.245	3.96 ± 0.752	3.97 ± 0.541	3.86 ± 0.029
Liver/BW ratio	0.04 ± 0.005	0.04 ± 0.001	0.04 ± 0.004	0.04 ± 0.009
Kidney/BW ratio	0.01 ± 0.001	0.01 ± 0.001	0.01 ± 0.001	0.01 ± 0.001
Spleen/BW ratio	0.003 ± 0.000	0.003 ± 0.000	0.003 ± 0.000	0.003 ± 0.000
Glucose (mmol/L)	5.50 ± 0.99	5.40 ± 0.07	5.13 ± 0.76	5.00 ± 0.41
ALT (U/L)	53.68 ± 3.32	44.62 ± 1.83*^#^	44.33 ± 4.76*	43.55 ± 2.21*^#^
ALP (U/L)	72.33 ± 3.84	72.67 ± 3.37	79.67 ± 4.80	78.83 ± 1.89
AST (U/L)	103.97 ± 4.15	108.32 ± 2.10	108.04 ± 2.93	107.21 ± 5.83
Creatinine (μmol/L)	43.50 ± 0.40	42.00 ± 0.40	43.00 ± 0.95	42.50 ± 0.21

*and ^#^indicates a significant difference compared with the normal control group at *p* < 0.05 and *p* < 0.001

### In vivo antioxidant profile

**Fig. 1:**
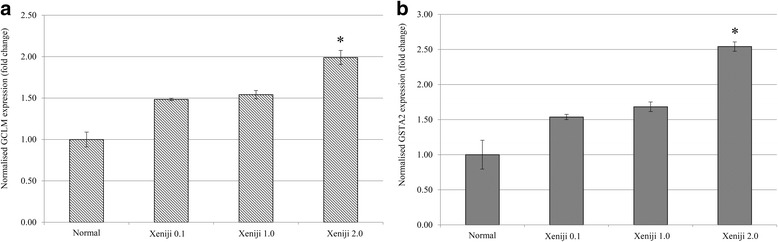
Differential expression of (**a**) GCLM and (**b**) GSTA2 genes related to Nrf-2 antioxidant pathway on normal and Xeniji (0.1, 1 and 2 g/kg body weight) treated mice. The expression of target genes (±SEM) were normalised to β-actin and the normal group was used as control for comparison. Fold change >2 comparing to normal control group was considered as significant*

**Fig. 2:**
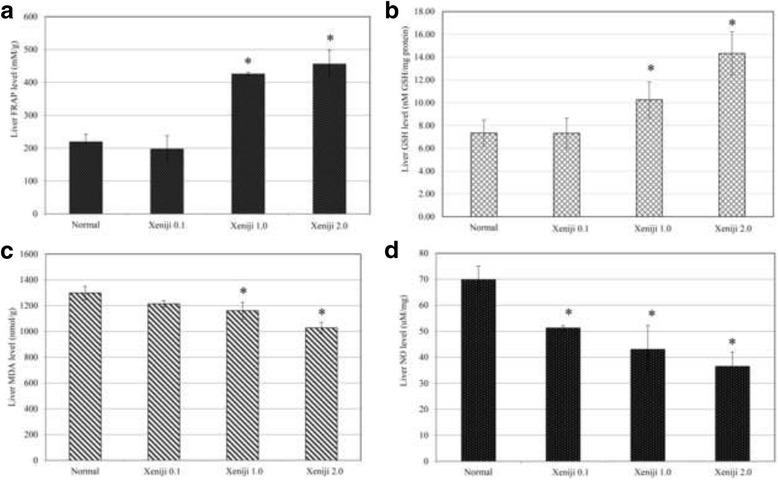
**a** Total antioxidant capacity by quantified FRAP level; **b** GSH level; **c** lipid peroxidation MDA level and **d** NO level in liver of normal and Xeniji (0.1, 1 and 2 g/kg body weight) treated mice. *indicates a significant difference compared with the normal control group, *p* < 0.05

Mice from subchronic toxicity were also subjected to antioxidant level detection to understand the benefit of Xeniji treatment in promoting antioxidant defence in the healthy subjects. Based on the qRT-PCR assay, all Xeniji treated mice were recorded with higher expression level of GCLM and GSTA2 comparing to normal control mice. However, comparing among all the tested concentration, only Xeniji 2.0 treatment significantly (>2 fold change) upregulated expression of both GCLM and GSTA2 genes in the liver (Fig. 1a and b). Overexpression of GCLM and GSTA2 were correlated to higher antioxidant level indicated by higher level of liver GSH (Fig. 2b) and FRAP activity (Fig. 2a) of Xeniji treated mice, particularly at concentration of 1 and 2 g/kg bw. Higher level of liver antioxidant at Xeniji 1.0 and 2.0 treated mice also contributed to reduction of oxidative stress in the liver indicating by the lower level of lipid peroxidation (Fig. 2c) and nitric oxide (Fig. 2d).

### In vivo cytokine profile

**Fig. 3:**
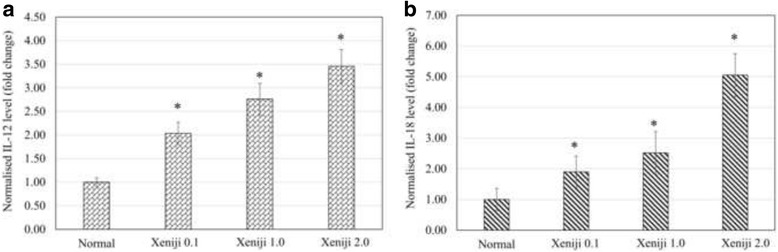
Differential expression of (**a**) IL-12 and (**b**) IL-18 genes in spleen of normal and Xeniji (0.1, 1 and 2 g/kg body weight) treated mice. The expression of target genes (±SEM) were normalised to β-actin and the normal group was used as control for comparison. Fold change >2 comparing to normal control group was considered as significant*

**Fig. 4:**
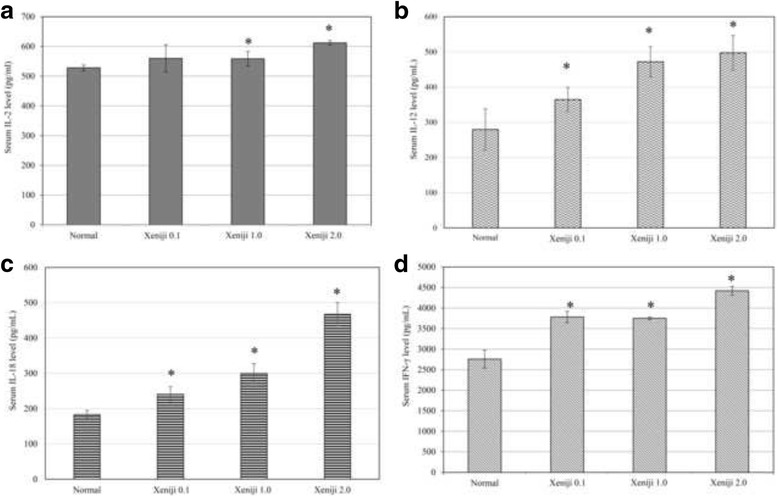
Serum (**a**) IL-2; (**b**) IL-12; (**c**) IL-18 and (**d**) IFN-γ level in liver of normal and Xeniji (0.1, 1 and 2 g/kg body weight) treated mice. *indicates a significant difference compared with the normal control group, *p* < 0.05

Besides the antioxidant level, immunity of the Xeniji treated healthy mice was also determined by evaluating the serum cytokines level, spleen T and NK cell population and cytotoxicity of splenocytes on Yac-1 cells. Expression IL-12 and IL-18 cytokine genes in the spleen were evaluated using qRT-PCR. All concentration of Xeniji was able to significantly (>2 fold changes) enhanced IL-12 (Fig. 3a) and IL-18 (Fig. 3b) expression in the spleen as compared to the normal healthy mice. Upregulation of IL-12 and IL-18 gene expression have contributed to the higher level of serum IL-2, IL-12, IL-18 and IFN-γ for the Xeniji 1.0 and 2.0 treated mice (Fig. 4).

### Immunophenotyping of CD3^+^CD4^+^, CD3^+^CD8^+^ and NK1.1

**Fig. 5:**
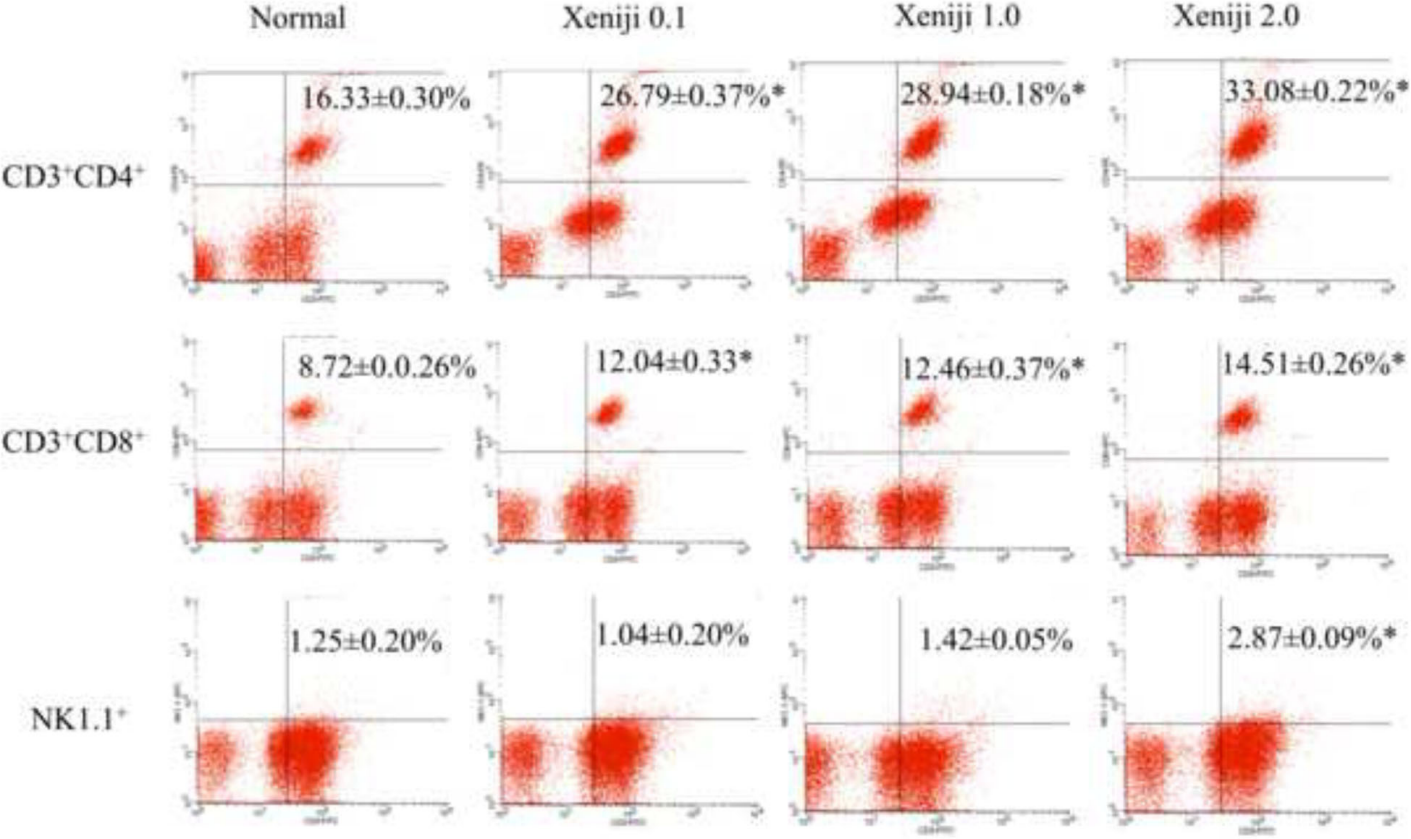
Immunophenotyping of splenic CD3^+^CD4^+^, CD3^+^CD8^+^ and CD3^+^NK1.1^+^ population by flow cytometer. * indicates a significant difference compared with the normal control group, *p* < 0.05

Population of T and NK cells in the spleen of normal and Xeniji treated mice were evaluated using flow cytometer. Mice treated with at all tested concentration of Xeniji were recorded with higher percentage of both CD3^+^CD4^+^ and CD3^+^CD8^+^ population, in dosage dependent manner. On the other hand, only Xeniji 2.0 was able to significantly increased NK cells population comparing to the normal control (Fig. 5).

### Cocultivation of splenocytes with YAC-1 cells

**Fig. 6:**
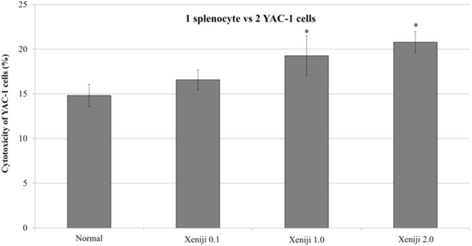
Cytotoxicity of splenocytes on YAC-1 cells at ratio 2 to 1. * indicates a significant difference compared with the normal control group, *p* < 0.05

YAC-1 cells are known to sensitive to cytolytic activity of NK cells. To further validate the immunostimulatory effect of Xeniji, splenocytes harvested from untreated and Xeniji treated mice were co-culture with YAC-1 cells at ratio 2:1. When compared to normal mice, only splenocytes harvested from mice treated with Xeniji 1.0 and 2.0 significantly (*p* < 0.05) possessed higher cytotoxicity on YAC-1 cells (Fig. 6).

## Discussion

Fruits and vegetables are good source of nutrients and phytochemicals, while fermentation is one of the important method to preserve fruits and vegetables that generally have short shelf life. These fermented fruits and vegetables were reported as source of polyphenols [22], which contributed to various bioactivities including antioxidant and immune stimulation [23]. Lactic acid bacteria fermented fruits and vegetables are natural product that commonly consumed in Japan [6, 8]. In Japan, fermented food paste that produced by fermenting fruits, vegetables and herbs has been commonly consumed as functional food [6, 8]. Nutritional value and bioactivities of some of the fermented food pastes have been previously reported [6, 8]. Xeniji is a fermented food paste that produced by fermenting fruits, herbs, mushroom and vegetables. Studies using other fermented food paste [6, 8] cannot directly represent the nutritional value, bioactivities and safety of all fermented foods as different ingredients and microbes used in the fermentation contributed to different level of nutrient and bioactivities of different fermented products [9]. Although fermented foods are generally considered safe, they may still associated with some risk factors [24]. In addition, over-consumption of polyphenols that commonly available in fermented foods may also cause some mild adverse effects [2]. Thus, it is important to evaluate the nutritional value, toxicity and in vivo antioxidant and immunomodulatory effects of Xeniji, which were yet to be determined.

Xeniji was rich in total phenolic and organic acids particularly citric acid, which is similar with the nutritional profile of another type of fermented food, plant based paste [6]. Fruits and vegetables were known as source of polyphenol antioxidant and citric acid [25, 26]. Thus, use of multiple fruits and vegetables as ingredient for fermentation contributed to high content of polyphenol and citric acid that directly contribute to the antioxidant effect of Xeniji. Besides antioxidant, Xeniji was also recorded with high content of essential amino acids and vitamins particularly β-carotene. Fermentation was known to reduce non-digestible carbohydrates and enrich the content of various nutrients including essential amino acids [27]. More particularly, cereal, which was one of the ingredients in fermentation of Xeniji, was able to digest by lactic acid bacteria to synthesise essential amino acids and vitamins [28]. In addition, GABA, which is the nonprotein amino acid that present in some medicinal plant such as *Zingiber officinale* [29], which was one of the food ingredient used in the preparation of Xeniji was also detected. Besides from the source plant, lactic acid that used in the fermentation of Xeniji was also reported as major GABA producing microorganism [30]. As Xeniji was rich in antioxidant and nutrient, the safety and efficacy were further tested in vivo.

Acute and subchronic toxicity evaluation are the studies that recommended by Organization of Economic Co-operation and Development (OECD) guideline for testing on the toxicity of chemicals including food. In this experiment, rate of mortality, body weight change, and serum liver enzyme markers were the parameters that commonly measured [31]. In this study, Xeniji did not observed with mortality and abnormality in both acute and subchronic toxicity up to 5 and 2 g/kg body weight, respectively. In addition, no significant change was observed in the body weight, organ weight and serum liver enzyme profile. This result indicated that Xeniji did not caused acute and subchronic toxic effect up to 5 and 2 g/kg body weight, respectively. These results have supported that lactic acid bacteria and yeast fermented fruits and vegetables are generally safe to be consumed [24, 32].

In vitro antioxidant study has shown that Xeniji possess antioxidant effect similar to another fermented foods PBP [6], which was contributed by the present of polyphenols and citric acid. In this study, the antioxidant level in the liver of normal and Xeniji treated mice were evaluated by FRAP, GSH, MDA and NO quantifications. Liver harvested from Xeniji treated mice were recorded with higher level of FRAP total antioxidant associated with reduction of MDA and NO levels in dosage dependent level. Glutamate-cysteine ligase complex modifier subunit (GCLM) and glutathione S-transferases 2 (GSTA2) were genes under nuclear factor erythroid 2-related factor 2 (Nrf2) antioxidant pathways. These genes involved in production and utilisation of GSH [33]. In this study, expression of both GCLM and GSTA2 genes were upregulated in the liver of Xeniji treated mice particularly at concentration 2 μg/mL indicating that Xeniji was effective in stimulating generation and utilisation of GSH, which subsequently contributed to the promotion of total antioxidant activity in the liver. This effect may further contribute to the inhibition of lipid peroxidation and NO as previous report has shown that GSH also help to suppresse the reactive oxygen species and reactive nitrogen species [34].

Besides activation of antioxidant level, fermented foods were also found with immunomodulatory effect [35] including activation on cytotoxicity of mice natural killer (NK) and lymphokine-activated killer (LAK) cells [12]. In this study, the immunomodulatory effect of normal and Xeniji treated mice were tested by measuring T/NK cells population, serum cytokines level and splenocyte cytotoxicity on YAC-1 cells. Xeniji treatments were found with significantly increase of both splenic helper (CD3^+^CD4^+^) and cytolytic (CD3^+^CD8^+^) T lymphocytes population in dosage dependent manner. On the other hand, population of splenic NK cells were only found significantly increased in the Xeniji 2.0 treated mice. This result show that Xeniji mainly activating T lymphocyte. Moreover, Xeniji treated mice were also found with higher level of serum IL-12, IL-18 and IFN-γ. IL-12 and IL-18 are cytokines that produced mainly by antigen presenting cells and macrophages [36]. IL-12 and IL-18 synergistically promote proliferation and IFN-γ production of both CD4 and CD8 T cells [36, 37]. Our results show that higher population of CD4 and CD8 T cells in Xeniji treated mice maybe contributed mainly by the activation of IL-12 and IL-18 production. On the other hand, Xeniji 1.0 and 2.0 were found significantly upregulated the serum IL-2 level. IL-2 is cytokine that produced mainly by CD4^+^ T cells and activates cytolytic activity of NK cells [38]. In this study, splenocyte harvested from mice treated with Xeniji 1.0 and 2.0 were recorded with significantly higher level of cytotoxicity on YAC-1 cells, which is sensitive to killing of NK cells [39]. Activation of NK cells cytotoxicity in Xeniji 1.0 and 2.0 treated mice maybe directly contributed by the higher level of IL-2.

## Conclusions

This study has shown that Xeniji was rich in β-carotene, phytonadione, polyphenol, citric acid and essential amino acid that enhance the antioxidant level and strengthen immunity in dosage dependent manner without causing acute and subchronic toxicity. As Xenji enhance antioxidant and immunity of healthy subjects, it maybe a potential nutraceutical agent to prevent and ameliorate infectious or oxidative induced diseases.

## Additional files

Additional file 1: Figure S1. UPLC profile of Xeniji water extract. (TIFF 7711 kb)

Additional file 2: Figure S2. Standard curve and melt curve of qPCR targets. (TIFF 14749 kb)

## Abbreviations

ALP: Alkaline phosphatase
ALT: Alanine transaminase
ANOVA: One-way analysis of variance
AST: Aspartate aminotransferase
BW: Body weight
GABA: Gamma amino butyric acid
GCLM: Glutamate-cysteine ligase complex modifier subunit
GSTA2: Glutathione S-transferases2
IACUC: Institution Animal Care and Use Committee
LAK: Lymphokine-activated killer
MDA: Malondialdehyde
NK: Natural killer
NO: Nitric oxide
Nrf2: Nuclear factor erythroid 2-related factor 2
PBP: Plant-based fermented paste
RT-qPCR: Reverse transcriptase-quantitative real time PCR
UT: untreated
