# Supplementary figures and images for: In vitro characterization and in vivo toxicity, antioxidant and immunomodulatory effect of fermented foods; Xeniji™
Source: BMC Complement Altern Med. 2017 Jun 30;17:344. doi: 10.1186/s12906-017-1845-6 (PMC5493119; doi:10.1186/s12906-017-1845-6)

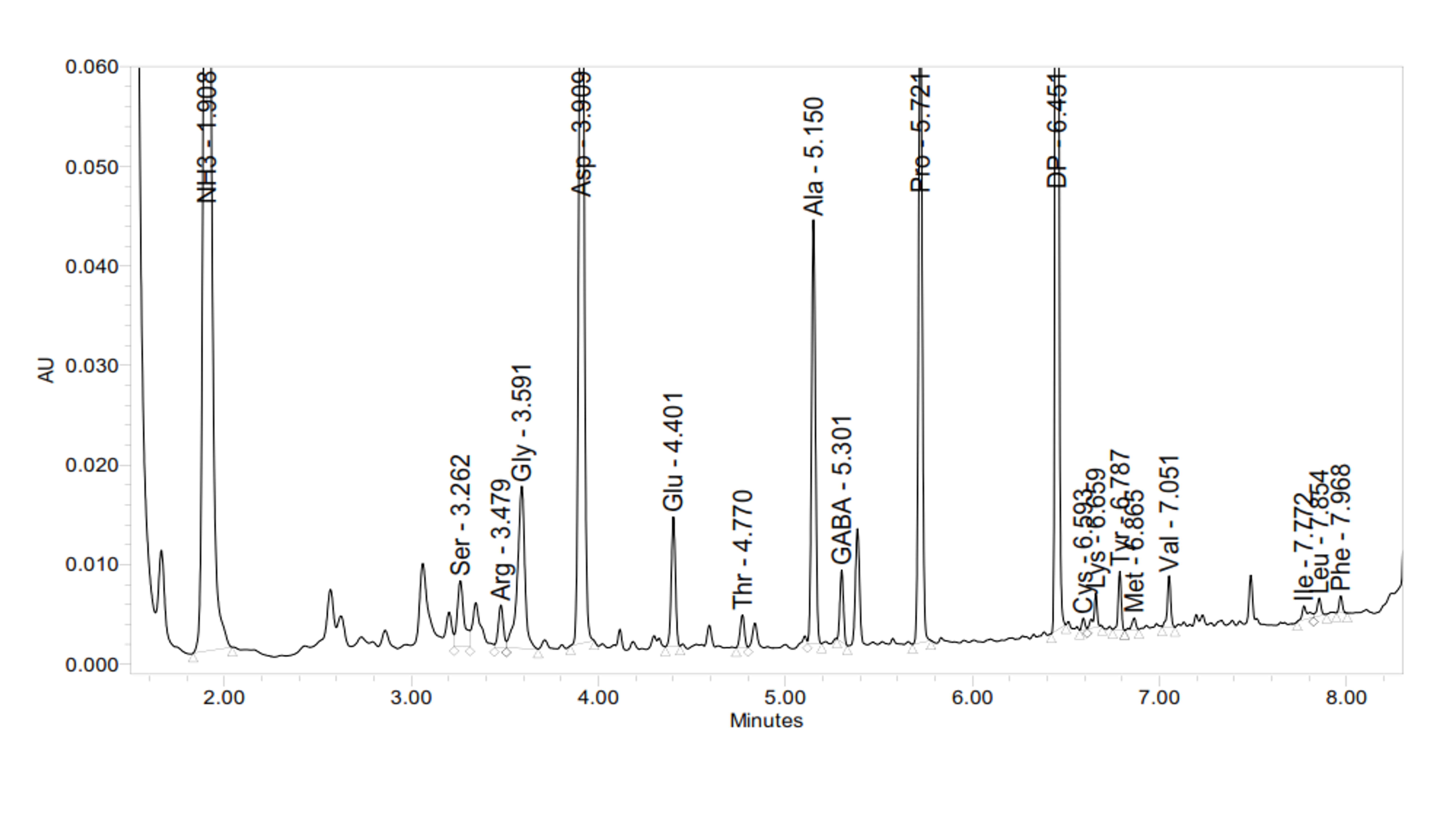

Supplement: Supplementary file 1 — UPLC profile of Xeniji water extract. (TIFF 7711 kb) [file 12906_2017_1845_MOESM1_ESM.tif]

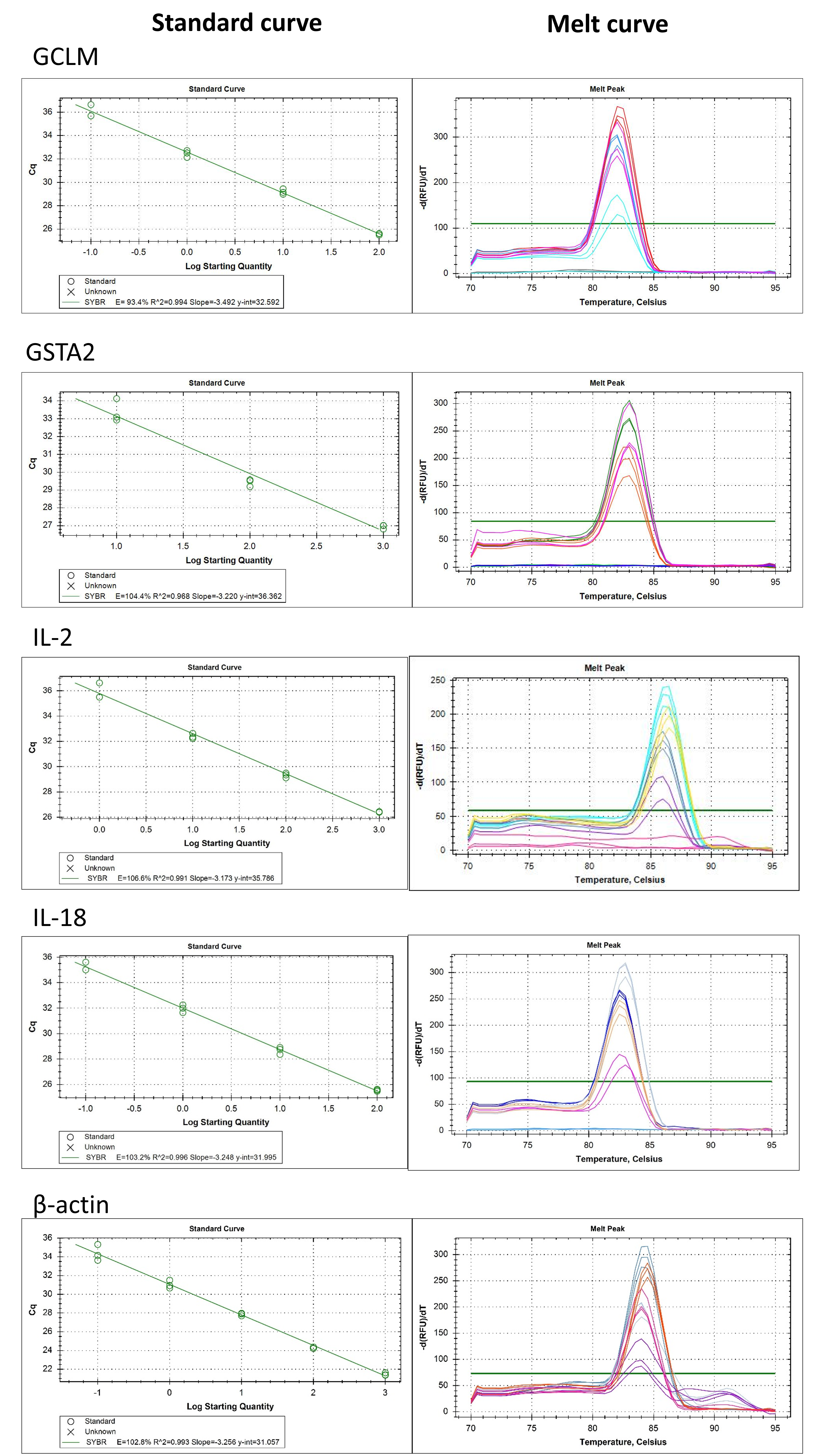

Supplement: Supplementary file 2 — Standard curve and melt curve of qPCR targets. (TIFF 14749 kb) [file 12906_2017_1845_MOESM2_ESM.tif]
